# Molecular MRI monitoring of cyclodextrin therapy in murine abdominal aortic aneurysms

**DOI:** 10.1038/s41598-026-61318-8

**Published:** 2026-07-20

**Authors:** Jennifer L. Heyl, Dilyana B. Mangarova, Jana Möckel, Jennifer Mein, David Hingst, Uwe Karst, René M. Botnar, Lisa C. Adams, Marcus R. Makowski, Federico Collettini, Matthias Taupitz, Christa Thöne-Reineke, Bernd Hamm, Avan Kader, Julia Brangsch

**Affiliations:** 1 Department of Radiology, Charité – Universitätsmedizin Berlin, corporate member of Freie Universität Berlin and Humboldt-Universität zu Berlin, Charitéplatz 1, 10117 Berlin, Germany; 2 Department of Veterinary Medicine, Institute of Animal Welfare, Animal Behavior and Laboratory Animal Science, Freie Universität Berlin, Königsweg 67, 14163 Berlin, Germany; 3 Institute of Inorganic and Analytical Chemistry, University of Münster, Corrensstr. 48, 48149 Münster, Germany; 4 School of Biomedical Engineering and Imaging Sciences, King’s College London, St Thomas’ Hospital, Westminster Bridge Road, London, SE1 7EH UK; 5 Institute for Biological and Medical Engineering, Pontificia Universidad Católica de Chile, Avda.Vicuña Mackenna 4860, Macul, Santiago, 7820436 Chile; 6 School of Engineering, Pontificia Universidad Católica de Chile, Avda.Vicuña Mackenna 4860, Macul, Santiago, 7820436 Chile; 7 Millennium Institute for Intelligent Healthcare Engineering, Avda.Vicuña Mackenna 4860, Macul, Santiago, 7820436 Chile; 8 Institute for Advanced Study, Technical University of Munich, Lichtenbergstraße 2a, 85748 Garching, Germany; 9 Department of Diagnostic and Interventional Radiology, Technical University of Munich, Ismaninger Str. 22, 81675 Munich, Germany

**Keywords:** Molecular imaging, Aneurysm, Magnetic resonance imaging, Therapy monitoring, Cardiology, Diseases, Medical research

## Abstract

Abdominal aortic aneurysms (AAAs) are characterized by progressive extracellular matrix (ECM) degradation and inflammation of the aortic wall. 2-Hydroxypropyl-β-cyclodextrin (cyclodextrin) has shown potential in attenuating AAA progression via activation of transcription factor EB. This study evaluated whether molecular magnetic resonance imaging (MRI) enables non-invasive monitoring of therapeutic effects in a murine AAA model. Thirty-two male apolipoprotein-E knockout mice with angiotensin II–induced AAAs received either cyclodextrin (*n* = 8) or saline (*n* = 10) for three weeks. A dual molecular MRI approach was applied using a gadolinium-based elastin-specific probe to assess ECM integrity and ultrasmall superparamagnetic iron oxide particles (USPIO, iron oxide particles) to evaluate macrophage-driven inflammation. MRI was performed at pre-treatment baseline and at 2 and 3 weeks after treatment initiation. Cyclodextrin-treated animals demonstrated significantly smaller aortic cross-sectional areas (2.31 ± 0.36 mm² vs. 2.96 ± 0.62 mm²; *p* = 0.018) and lower elastin-specific signal enhancement (1.56 ± 0.26 vs. 2.15 ± 0.69; *p* = 0.038), indicating preserved ECM integrity. In contrast, no significant differences in iron oxide particles-related signal changes were observed between groups (*p* = 0.441), consistent with histological and molecular findings. This study shows that elastin-specific molecular imaging non-invasively monitors cyclodextrin’s effects in AAA, whereas iron oxide particle imaging revealed no significant in vivo signal changes, consistent with ex vivo findings and indicating limited detectable inflammatory modulation associated with cyclodextrin treatment under the conditions of this study.

**Supplementary Information:**

The online version contains supplementary material available at 10.1038/s41598-026-61318-8.

## Introduction

Abdominal aortic aneurysms (AAAs) are a prevalent and potentially fatal cardiovascular condition, accounting for approximately 160,000 deaths worldwide in 2023.^1^ Defined as a dilation of the abdominal aorta ≥ 1.5 times its physiological diameter, AAAs are typically asymptomatic until rupture, which carries a mortality rate of 85–90%.^2–4^ The overall prevalence among individuals aged 30–79 years is approximately 0.9% (in both sexes), with males – particularly over 55 – at significantly higher risk.^3,5,6^.

Ultrasound is the primary modality for AAA diagnosis and routine monitoring because of its high sensitivity and wide availability.^3,7^ In addition, cardiovascular computed tomography (CT) and magnetic resonance imaging (MRI) are used for anatomical assessment and imaging.^3,7^ However, these imaging modalities lack information about aortic wall composition and biological activity, restricting their ability to assess therapeutic effects. In contrast, molecular MRI uniquely combines morphological imaging with visualization of biochemical processes through targeted probes.^8^ This technique allows not only detailed insight into disease mechanisms but also holds great promise for non-invasive therapy monitoring by visualizing treatment-induced changes in tissue composition.

AAA pathogenesis involves extracellular matrix (ECM) degradation and chronic inflammation, primarily driven by macrophages that release cytokines and matrix metalloproteinases (MMPs) which degrade elastin and collagen.^9,10^ The resulting wall weakening promotes aneurysm expansion and rupture. These molecular changes offer potential targets for advanced imaging in preclinical studies. Previous MRI studies demonstrated the ability to simultaneously utilize two distinct molecular probes in a single imaging session, to maximize the diagnostic information obtained and predict AAA rupture.^11,12^ These pathophysiological features not only drive AAA progression but also serve as quantifiable biomarkers for therapeutic monitoring using molecular MRI.

Medical management of AAAs aims to delay progression and reduce rupture risk.^5^ While surgical repair in humans is indicated for aneurysms > 5.5 cm in diameter or those showing rapid growth, current medical strategies include blood pressure control, statins, smoking cessation, and various pharmacological agents being tested in ongoing clinical trials.^3,5,13^ A promising candidate is 2-hydroxypropyl-β-cyclodextrin (cyclodextrin), which has been shown to inhibit AAA progression by activating transcription factor EB (TFEB) that protects vascular smooth muscle cells (VSMCs) and reduces elastin degradation and inflammation.^14,15^ However, non-invasive tools to assess the biological response to such therapy are not routinely established. This highlights the need for imaging approaches that can monitor treatment effects in vivo.

In this study, we investigated the potential of molecular MRI to monitor cyclodextrin therapy in a murine AAA model using an elastin-specific gadolinium probe to assess ECM integrity and iron oxide particles to evaluate macrophage-mediated inflammation. The elastin-specific probe has been previously employed in various studies investigating abdominal aortic aneurysms, atherosclerosis and competition experiments.^12,16,17^ The probe binds to elastin fibers, leading to increased local gadolinium accumulation and, consequently, higher signal intensity. As a result, regions with higher elastin content exhibit a higher contrast-to-noise ratio (CNR). This includes both preserved elastin fibers and compensatory, often disorganized, elastin neoformation associated with extracellular matrix remodeling. Based on prior findings, we hypothesize that cyclodextrin treatment will result in reduced ECM degradation and thereby attenuate compensatory elastin neoformation, which is expected to be reflected by a lower contrast-to-noise ratio (CNR) increase in the treated group. For the assessment of inflammation, ferumoxytol was used as an ultrasmall superparamagnetic iron oxide nanoparticle (USPIO) agent. Due to the particle size (approximately 30 nm), these nanoparticles are phagocytized by cells of the mononuclear phagocyte system.^18^ Inflamed tissue shows increased uptake of iron oxide particles by macrophages, leading to a greater relative signal reduction compared to non-inflamed tissue. Given the suggested anti-inflammatory effects associated with TFEB activation, we further hypothesize that treatment will lead to reduced macrophage activity, which will be reflected by a reduced uptake and signal change of iron oxide particles in the treated group. The combination of the elastin-specific probe and iron oxide particles has been previously examined in a dual probe approach in an AAA mouse model.^12^.

We aimed to determine whether cyclodextrin therapy–induced changes in elastin degradation and inflammation can be reliably evaluated and quantified using dual (structural and inflammatory) molecular MRI.

## Results

### Morphological MR measurements

**Fig. 1:**
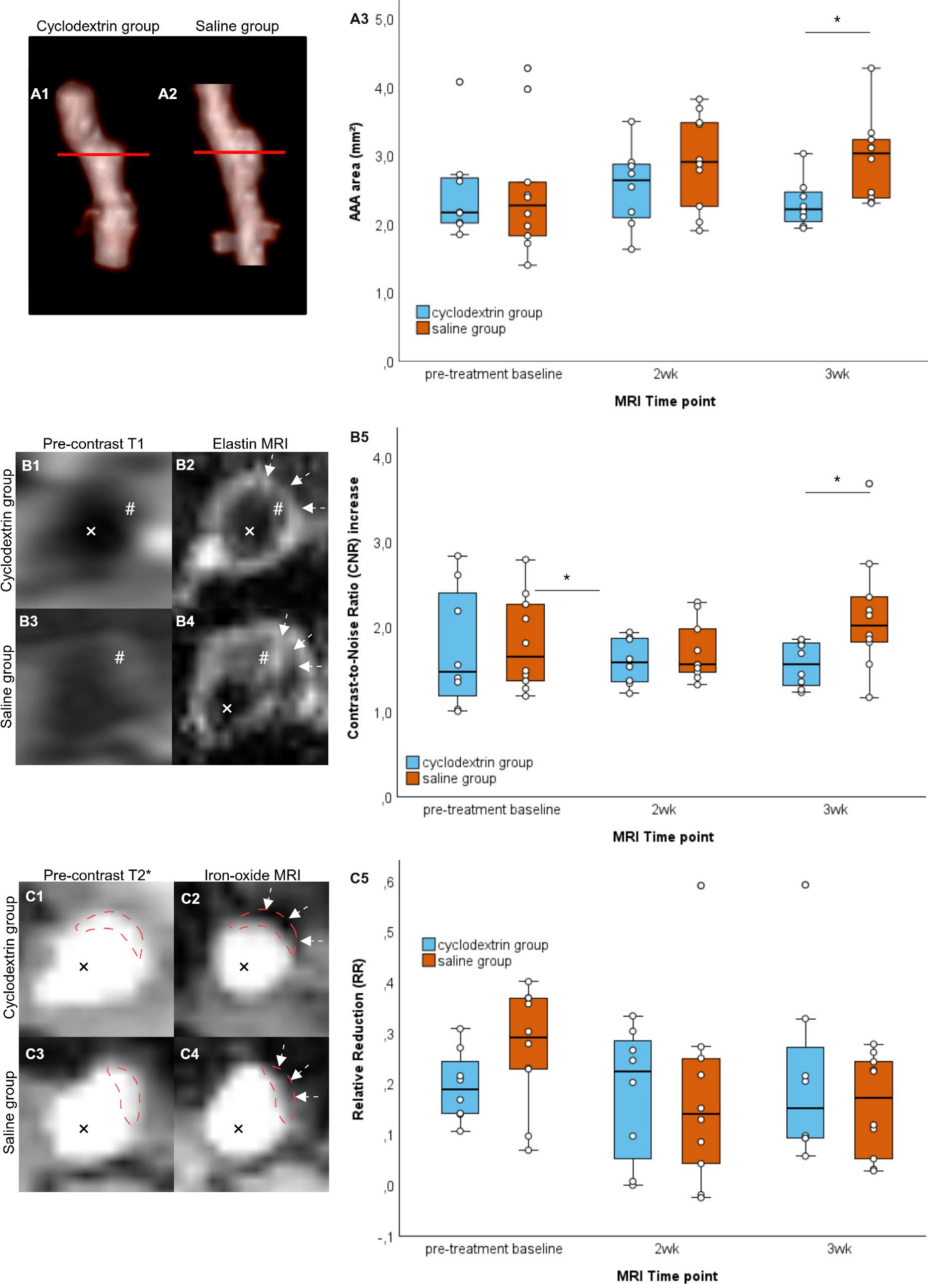
In vivo molecular MRI measurements of aortic morphology, elastin content, and inflammation. **A**, Abdominal aortic aneurysm (AAA) morphology could be visualized using 3D Models of both groups, with the red lines marking the AAA cross-section (**A1**, **A2**). **A3**: Cross-sectional AAA area was comparable between groups at pre-treatment baseline-MRI. After 3 weeks of treatment, the cyclodextrin-treated group (*n* = 8 biological replicates) showed a significantly smaller cross-sectional aortic area compared to saline controls (*n* = 10 biological replicates). Statistical analysis was performed using a two-tailed unpaired t-test: *p < 0.05 (*p* = 0.018). **B**, As shown in pre- and post-contrast MRI images (**B1**, **B2**, **B3**, and **B4**) the elastin-specific MR probe led to a contrast-to-noise ratio (CNR) increase in both groups at all timepoints (**B5**). At 3wk-MRI, the cyclodextrin group (*n* = 8 biological replicates) showed a significantly lower CNR increase than controls (*n* = 10 biological replicates). For each animal, three MRI slices were analysed and averaged prior to statistical analysis. Statistical analysis was performed using a two-tailed unpaired t-test: *p < 0.05 (*p* = 0.038). **C**, T2*-weighted MRI images before and after iron oxide particle administration (**C1**, **C2**, **C3**, and **C4**) allowed visualization of signal changes associated with iron uptake. However, quantitative analysis revealed no significant differences in T2*-weighted signal reduction between groups at any timepoint (**C5**), (cyclodextrin group: *n* = 8 biological replicates, saline group: *n* = 10 biological replicates). For each animal, three MRI slices were analysed and averaged prior to statistical analysis. Statistical analysis was performed using a two-tailed unpaired t-test: ns (*p* = 0.441). Statistical significance: *ns = not significant (p ≥ 0.05)*, * *p* < 0.05, ** *p* < 0.01, *** *p* < 0.001. # indicates a thrombus; × indicates the lumen; Arrows and dotted red line: area of signal change. Created in BioRender. Brangsch, J. (2026) https://BioRender.com/rikk7vl.

At the pre-treatment baseline MRI, initial aneurysm sizes were comparable between groups, with a cross-sectional AAA area of 2.48 ± 0.94 mm² in the cyclodextrin group (*n* = 8) and 2.46 ± 0.72 mm² in the saline group (*n* = 10), *p* = 0.964, d = 0.02 (Fig. 1A).

An intragroup AAA growth longitudinal analysis was performed and demonstrated that up to the final MRI (3wk-MRI), the cyclodextrin group showed non-significant increase in aortic area, with a trend toward reduction (2.31 ± 0.36 mm² at 3wk-MRI vs. pre-treatment baseline), *p* = 0.519, d = 0.24. The saline group exhibited a non-significant increase in AAA area (2.96 ± 0.62 mm² at 3wk-MRI vs. pre-treatment baseline), *p* = 0.054, d = 0.70 (Fig. 1A).

Direct comparison of both groups at 3wk-MRI (cyclodextrin group 2.31 ± 0.36 mm² vs. saline group 2.96 ± 0.62 mm²) revealed a significantly smaller cross-sectional aortic area in the cyclodextrin group compared to the control group, *p* = 0.018, d = 1.25 (Fig. 1A). The values from the 2-wk MRI scan can be found in Table 1.

**Table 1:** Representation of in vivo MRI measurements over time.

parameter	group	pre-treatment baseline-MRI	2wk-MRI	3wk-MRI
diameter AAA (mm)	cyclodextrin	1.88 ± 0.20	1.83 ± 0.15	1.73 ± 0.12
	saline	1.83 ± 0.25	2.03 ± 0.21	2.11 ± 0.25
area AAA (mm²)	cyclodextrin	2.48 ± 0.94	2.55 ± 0.59	2.31 ± 0.36
	saline	2.46 ± 0.72	2.93 ± 0.69	2.96 ± 0.62
circumference (mm)	cyclodextrin	5.62 ± 0.75	5.70 ± 0.65	5.46 ± 0.37
	saline	5.61 ± 0.99	6.15 ± 0.78	6.21 ± 0.65
CNR increase	cyclodextrin	1.75 ± 0.71	1.60 ± 0.27	1.56 ± 0.26
	saline	1.81 ± 0.55	1.71 ± 0.35	2.15 ± 0.69
RR	cyclodextrin	0.20 ± 0.07	0.18 ± 0.13	0.21 ± 0.18
	saline	0.27 ± 0.12	0.17 ± 0.18	0.16 ± 0.10

Aortic diameter was additionally assessed in all MRI scans, and the corresponding measurements at each time point are reported in Table 1.

### Elastin-specific MR Probe assessment

The elastin-specific molecular probe was investigated to assess elastin content in vivo. Following administration of the elastin-specific MR probe, CNR increased significantly at all timepoints, *p* < 0.001, d = 1.387.

At pre-treatment baseline MRI, the relative CNR increase (post-/pre-contrast) was not significantly different between both groups: 1.75 ± 0.71 (cyclodextrin group) and 1.81 ± 0.55 (saline group), *p* = 0.833, d = 0.10 (Fig. 1B). At final MRI (3wk-MRI), CNR increase was significantly lower in the cyclodextrin group (1.56 ± 0.26) than in the saline group (2.15 ± 0.69), *p* = 0.038, d = 1.07 (Fig. 1B), indicating a lower elastin content in the cyclodextrin group.

### Iron oxide-enhanced T2* MRI assessment

To assess inflammation, iron oxide particles were examined as macrophage markers in vivo. At pre-treatment baseline MRI, the relative T2* signal reduction did not differ significantly between groups: 0.20 ± 0.07 (cyclodextrin group) and 0.27 ± 0.12 (saline group), *p* = 0.123, d = 0.77 (Fig. 1C). Differences could be explained by random unequal expression of inflammation at the beginning of the study. Similarly, at 3wk-MRI, no significant difference was observed: 0.21 ± 0.18 (cyclodextrin group) and 0.16 ± 0.10 (saline group), *p* = 0.441, d = 0.38 (Fig. 1C), indicating similar content of macrophages.

### Ex vivo analysis and its correlation to in vivo MRI findings

Ex vivo AAA area measurements showed a strong correlation with in vivo MRI data (*r* = 0.667, *p* = 0.003, Supplementary Fig. 2A).

**Fig. 2:**
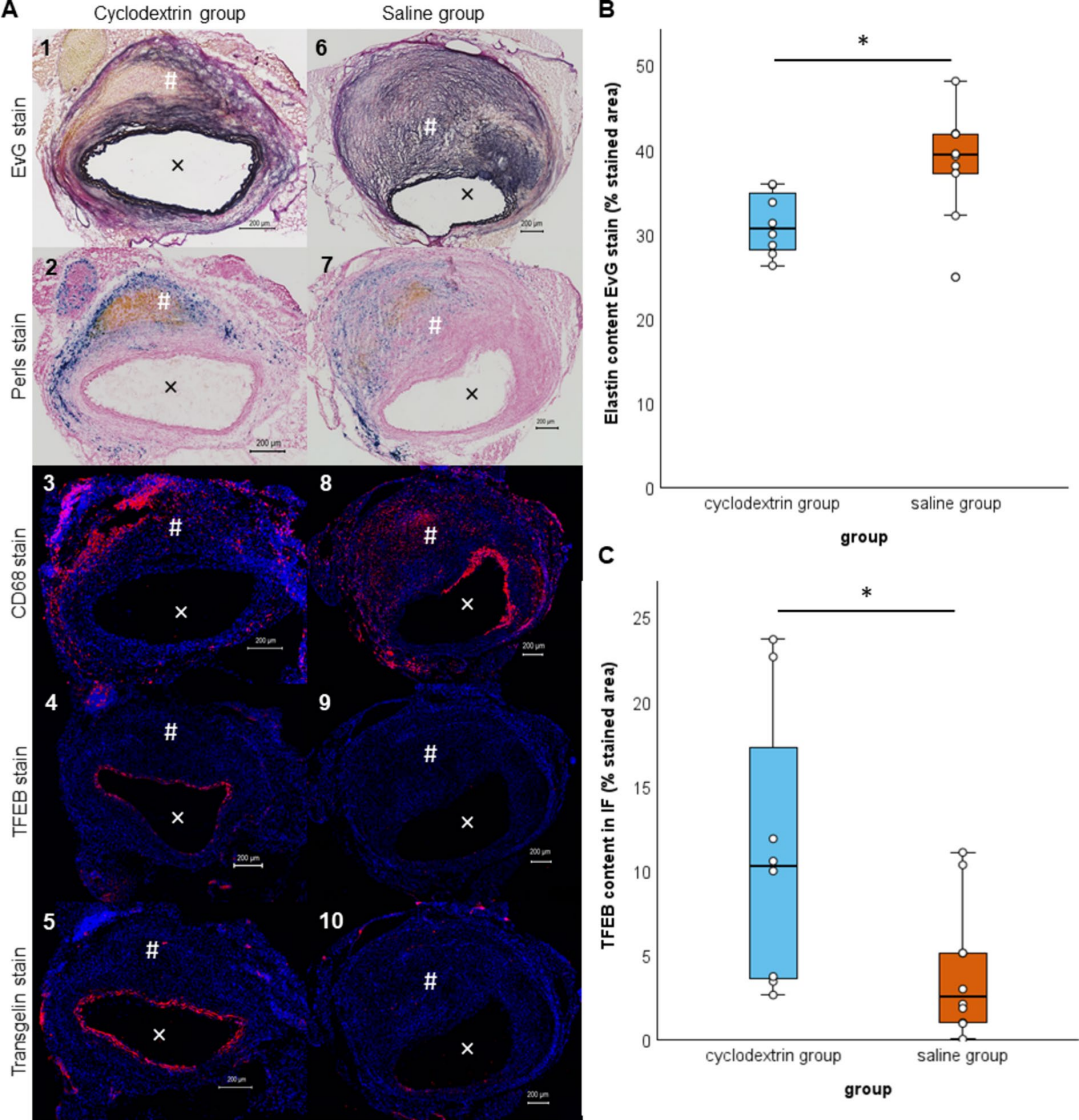
Ex vivo validation of molecular MRI findings. **A**, (**A1**, **A6**) Elastica van Gieson (EvG) staining revealed significantly reduced elastin content in the cyclodextrin group (*n* = 8 biological replicates) compared to saline controls (*n* = 10 biological replicates). Statistical analysis was performed using a two-tailed unpaired t-test: *p < 0.05 (*p* = 0.01). (**A2**, **A7**) Perls’ Prussian Blue staining showed no significant difference in iron deposits between groups (cyclodextrin group: *n* = 8 biological replicates, saline group: *n* = 10 biological replicates). Two-tailed unpaired t-test: ns (*p* = 0.988). (**A3**, **A8**) CD68 immunofluorescence indicated a non-significant trend toward reduced macrophage presence in the cyclodextrin group (cyclodextrin group: *n* = 8 biological replicates, saline group: *n* = 10 biological replicates). Two-tailed unpaired t-test: ns (*p* = 0.286). Immunofluorescence for transcription factor EB (TFEB) (**A4**, **A9**) and transgelin (**A5**, **A10**) revealed higher expression in the cyclodextrin group (cyclodextrin group: *n* = 8 biological replicates, saline group: *n* = 10 biological replicates). Two-tailed unpaired t-test: TFEB *p < 0.05 (*p* = 0.03) and transgelin ns (*p* = 0.113). **B**, Elastin content determined by EvG staining, expressed as the percentage of stained area; (cyclodextrin group: 31.27 ± 3.68% compared to the saline group: 38.59 ± 6.29%, two-tailed unpaired t-test: *p* = 0.01). **C**, The difference in TFEB content in immunofluorescence staining was statistically significant (cyclodextrin group: 11.28±8.28%, saline group: 4.09±3.89%, *p* = 0.03) (cyclodextrin group: *n* = 8 biological replicates, saline group: *n* = 10 biological replicates). Two-tailed unpaired t-test: *p < 0.05 (*p* = 0.03). Statistical significance: *ns = not significant (p* ≥ 0.05), * *p* < 0.05, ** *p* < 0.01, *** *p* < 0.001. *#* indicates a thrombus; × indicates the lumen; Scale bar: 200 μm.

Elastin Content Assessment: EvG staining revealed significantly reduced elastin content in the cyclodextrin group (31.27 ± 3.68%) compared to the saline group (38.59 ± 6.29%), *p* = 0.01, d = 1.38 (Fig. 2A1, A6, B). Ex vivo elastin values correlated well with in vivo CNR measurements (*r* = 0.695, *p* = 0.001, Supplementary Fig. 2B).

Iron Deposition and Macrophage Analysis: To detect and localize iron deposits, indicative of macrophage activity, histological sections were stained using Perls’ Prussian blue staining, which selectively highlights ferric iron as blue deposits within the tissue. Perls’ Prussian Blue staining showed no significant difference in iron deposits between groups: 1.72 ± 3.02% (cyclodextrin group) and 1.74 ± 2.76% (saline group), *p* = 0.988, d = 0.01 (Fig. 2A2, A7, Supplementary Fig. 1B). Macrophage presence was further evaluated using immunofluorescence staining for CD68, a surface marker specific for monocytes and macrophages. CD68 immunofluorescence indicated a non-significantly lower macrophage presence in cyclodextrin-treated animals (5.99 ± 5.88%) than in saline-treated animals (8.49 ± 3.66%), *p* = 0.286, d = 0.52 (Fig. 2A3, A8, Supplementary Fig. 1C). In vivo T2* signal reductions correlated significantly with both iron content (*r* = 0.737, *p* < 0.001) and CD68-positive cells (*r* = 0.699, *p* = 0.001; Supplementary Fig. 2C, D).

### Ex vivo analyses of cyclodextrin effects on transcription factor EB and transgelin

Cyclodextrin represents an activator of Transcription factor EB which therefore results in an inhibition of the apoptosis of VSMCs.^14^ Immunofluorescence analysis of histological sections demonstrated significantly higher expression of TFEB after cyclodextrin treatment (11.28 ± 8.28%) compared to saline treatment (4.09 ± 3.89%), *p* = 0.03, d = 1.13 (Fig. 2A4, A9, Fig. 2C). Transgelin, a VSMC marker, showed a non-significant increase in expression in the cyclodextrin group (13.05 ± 7.61%) compared to saline (7.72 ± 5.94%), *p* = 0.113, d = 0.79 (Fig. 2A5, A10, Supplementary Fig. 1A).

### Western blot analysis

**Fig. 3:**
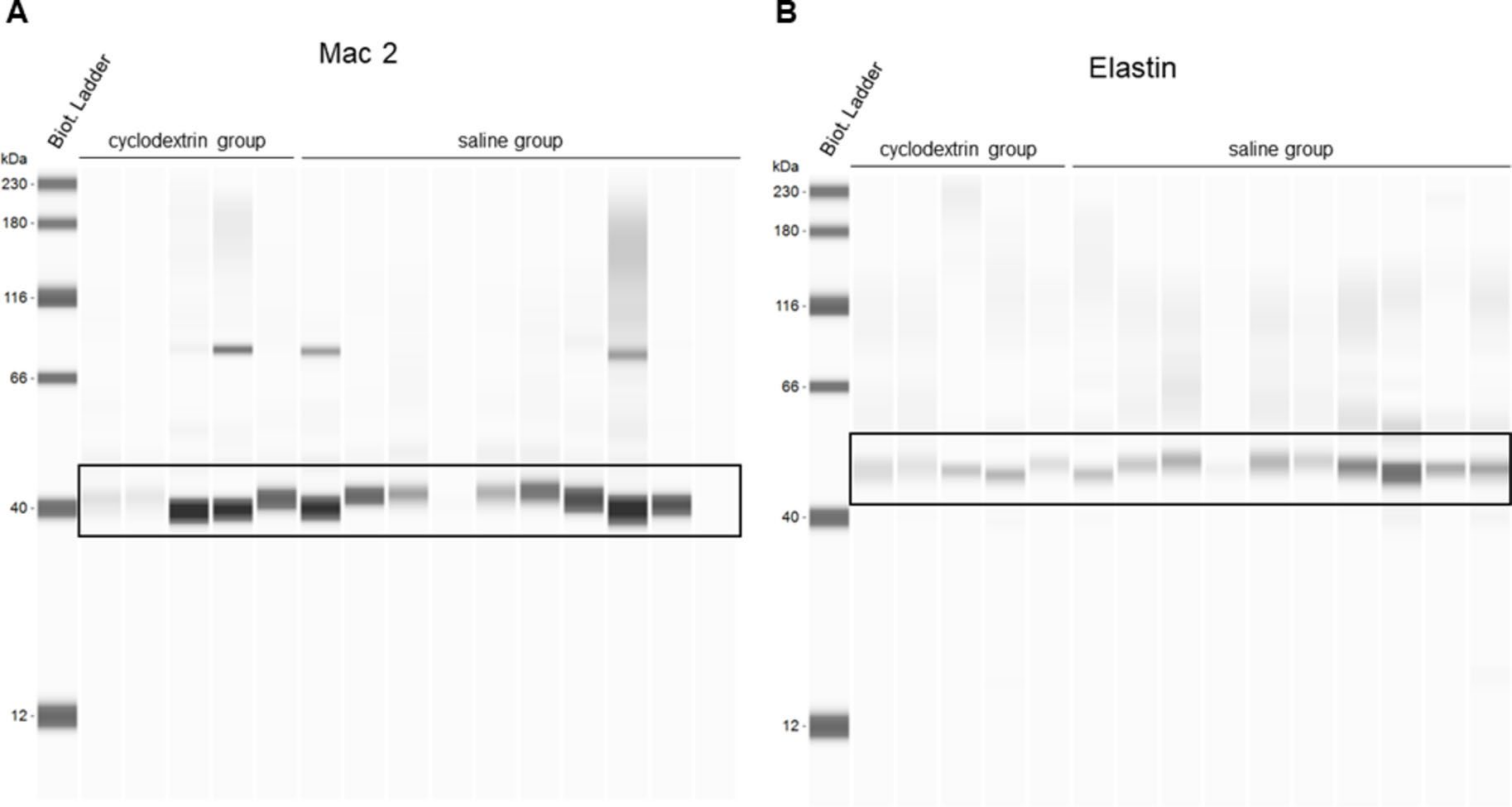
Western blot analysis. **A** ,Western blot analysis (cyclodextrin group: *n* = 5 biological replicates, saline group: *n* = 10 biological replicates) confirmed no significant difference in Mac2 (macrophage marker) content (cyclodextrin group: 1.38 × 10⁶ ± 0.87 × 10⁶, saline group: 2.35 × 10⁶ ± 1.29 × 10⁶, two-tailed unpaired t-test: *p* = 0.155), **B**, but significantly lower elastin content in cyclodextrin-treated animals (1.29 × 10⁶ ± 0.25 × 10⁶) than in the saline group (2.83 × 10⁶ ± 1.78 × 10⁶), Welch’s t-test: *p* = 0.025.

To further investigate extracellular matrix remodeling and inflammatory activity, Western blot analyses were conducted ex vivo to assess macrophage presence (Mac2) and elastin content. Mac2 was less abundant in cyclodextrin-treated mice (1.38 × 10⁶ ± 0.87 × 10⁶) than in saline-treated controls (2.35 × 10⁶ ± 1.29 × 10⁶), though this difference was not statistically significant, *p* = 0.155, d = 0.83 (Fig. 3A, Supplementary Fig. 1D). Western blotting confirmed significantly lower elastin levels in the cyclodextrin group (1.29 × 10⁶ ± 0.25 × 10⁶) compared to saline (2.83 × 10⁶ ± 1.78 × 10⁶), Welch’s t-test: *p* = 0.025, d = 1.03 (Fig. 3B, Supplementary Fig. 1E).

### Elemental analysis of aortic tissue

**Fig. 4:**
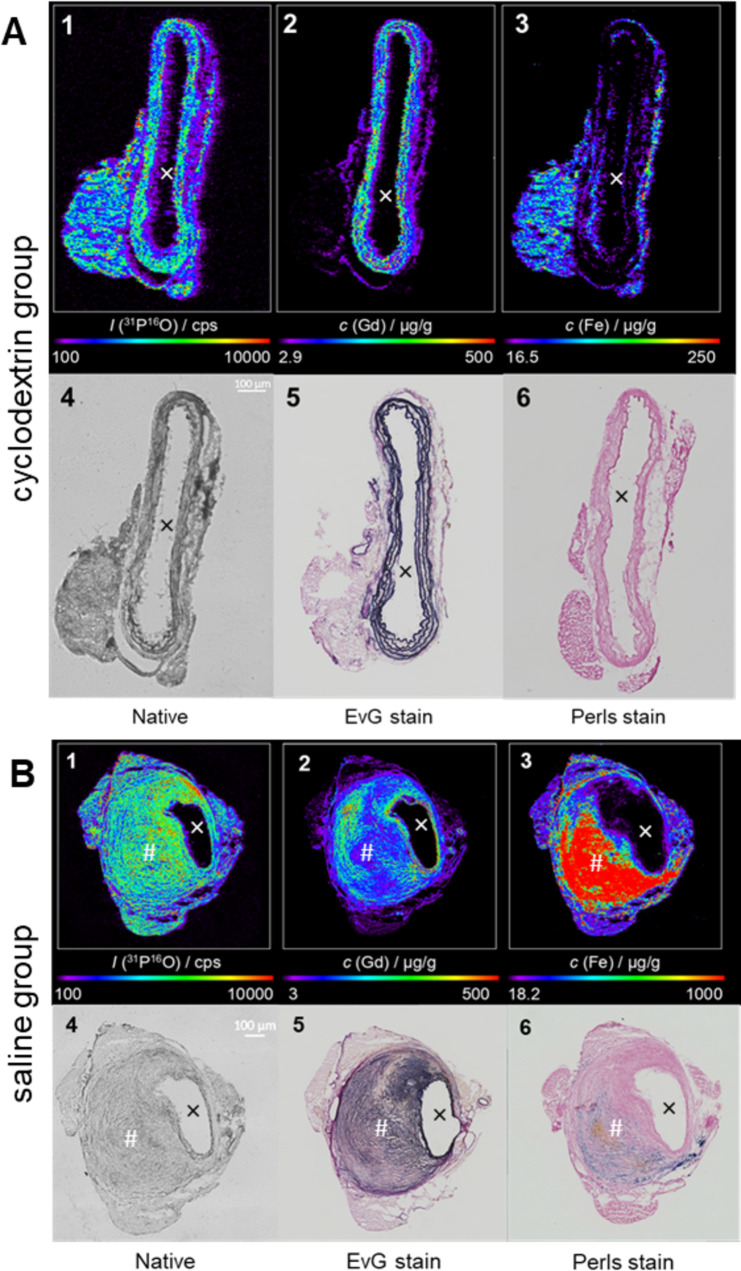
Elemental mapping of gadolinium and iron using LA-ICP-MS. LA-ICP-MS was used to visualize the distribution of gadolinium (Gd) and iron (Fe) in aortic cross-sections from cyclodextrin-(**A**) and saline-treated (**B**) mice (*n* = 3 biological replicates per group). To provide anatomical context for the AAA sections, phosphorus distribution was assessed (**A1**, **B1)**. **A4** and **B4** show the unstained reference. Gadolinium signals colocalized with elastin-rich regions, confirming specific probe accumulation in the aortic wall (**A2**, **A5** and **B2**,** B5**). Iron signals revealed localized particle accumulation, showing pronounced iron deposition in the thrombus and adjacent wall (**A3**, **A6** and **B3**,** B6**). #: thrombus; ×: lumen; scale bar: 100 μm.

To assess the spatial distribution of gadolinium from the elastin-specific MRI probe and iron from iron oxide nanoparticles, LA-ICP-MS was performed on aortic sections from each group (*n* = 3 per group) (Fig. 4). For visualization, the ^31^P^16^O^+^ signal was used to identify cell-rich regions (Fig. 4A1, B1), complemented by corresponding light microscopy images (Fig. 4A4, B4). Gadolinium signals qualitatively colocalized with regions rich in elastic fibers (EvG staining) within the aortic wall, confirming targeted probe accumulation in elastin-rich structures (Fig. 4A2, A5, B2, B5). Gadolinium concentrations measured by LA-ICP-MS showed a moderate correlation with the in vivo CNR increase at 3wk-MRI (*r* = 0.364, *p* = 0.478). In contrast, a strong correlation was observed between gadolinium content and ex vivo elastin quantification based on EvG-stained area (*r* = 0.931, *p* = 0.007), while a moderate correlation was found with elastin expression assessed by Western blot (*r* = 0.565, *p* = 0.242). The recorded iron signals showed colocalization with iron deposits identified by histological staining (Perls stain), providing evidence for the uptake of iron particles (Fig. 4A3, A6, B3, B6). Iron content measured by LA-ICP-MS demonstrated only a weak correlation with the in vivo relative signal reduction at 3wk-MRI (*r* = 0.162, *p* = 0.759). However, a strong correlation was observed with macrophage-associated markers, including CD68-positive area in immunofluorescence (*r* = 0.806, *p* = 0.053), and a moderate correlation with Mac-2 expression determined by Western blot (*r* = 0.413, *p* = 0.416).

## Discussion

This study demonstrates that an elastin-specific molecular MR probe can effectively monitor therapeutic responses to cyclodextrin treatment in a preclinical AAA model. In contrast, iron oxide particle-enhanced MRI appeared less sensitive under the conditions of this study for monitoring cyclodextrin therapy, as no significant differences in inflammatory activity could be detected. This observation is supported by immunofluorescence and histological analyses, although these findings should be interpreted with caution given the limited sample size and scope of the ex vivo assessments. These results highlight the importance of matching molecular imaging probes to monitor specific therapeutic mechanisms.

### Cyclodextrin as a therapeutic agent in AAA

Cyclodextrin demonstrated efficacy in attenuating AAA progression, with 8 out of 10 treated animals maintaining aneurysm growth below the 100% threshold of physiological aortic diameter expansion. This threshold was predefined as an inclusion/exclusion criterion for therapeutic success and was based in part on aortic diameter, a parameter commonly used in clinical and experimental practice. Mechanistically, cyclodextrin treatment was associated with increased activation of TFEB, which promotes vascular smooth muscle cell survival by suppressing apoptosis. This aligns with prior work by Lu et al. (2020)^14^, confirming TFEB’s role in stabilizing the aortic wall by limiting VSMC loss, which is a key event in aneurysm pathogenesis.^14^ Importantly, our findings extend previous preclinical work by demonstrating that cyclodextrin´s therapeutic effects can be monitored non-invasively using molecular MRI.

### Elastin-specific MRI for therapy monitoring

The progression of AAAs is characterized by enzymatic degradation of elastin and collagen by MMPs, followed by compensatory but disorganized elastin neosynthesis.^19,20^ The observed lower elastin content in the treated group may be explained by a reduction in compensatory elastin remodeling. By attenuating matrix degradation through TFEB activation, cyclodextrin may reduce the stimulus for excessive, disorganized elastin neosynthesis. Consequently, untreated aneurysms may exhibit higher apparent elastin levels due to ongoing degradation and compensatory accumulation, whereas treated aneurysms reflect a more stable vascular state with reduced remodeling activity. Given that cyclodextrin activates TFEB, elastin represents an ideal target for monitoring treatment response.^14^ The elastin-specific MR probe has previously demonstrated utility for detecting compensatory elastin remodeling during aneurysm formation and atherosclerosis.^17,21^ Our study expands on this by validating the elastin-specific probe´s value for monitoring AAA therapy-induced changes.

The significant correlation between in vivo CNR changes and ex vivo elastin content validates this approach. Cyclodextrin treatment leads to reduced MMP expression (particularly MMP-9) through TFEB activation, thereby mitigating elastin degradation.^14^ As a result, the maladaptive overproduction of elastin often seen in untreated aneurysms was attenuated, reflecting preserved structural integrity.^22^ Our results show that successful treatment resulted in reduced probe uptake, as cyclodextrin-treated animals exhibited lower elastin content compared to controls. This demonstrates that elastin-specific molecular MRI complements standard anatomical imaging by providing mechanistic insights into therapeutic efficacy at the molecular level.

No separate sham cohort without AAA induction was included in the present study. This decision was made to minimize animal usage, as the baseline behavior and specificity of the elastin-specific molecular probe in the AngII-induced AAA mouse model have already been established in previous studies.^12,17^ The present study therefore focused specifically on evaluating the ability of molecular MRI to monitor therapy-associated changes in already established aneurysms rather than reassessing probe specificity in non-aneurysmal tissue.

### Limitations of iron oxide particle imaging for monitoring cyclodextrin therapy effects

While inflammation is a key driver in AAA progression, facilitated by macrophage and T-cell infiltration, our results indicate that cyclodextrin’s therapeutic effects operate primarily through matrix remodeling rather than direct anti-inflammatory mechanisms.^22^ Previous studies have shown that cyclodextrin activates TFEB, which not only downregulates MMP-expression but also may exert protective effects against endothelial inflammation.^15^ Neither in vivo imaging with iron oxide particles nor ex vivo histology revealed differences in inflammatory burden between cyclodextrin group and the saline group, consistent with findings from Lu et al. (2020).^14^ These findings suggest that iron oxide particles may be suboptimal for monitoring therapies like cyclodextrin, which do not target inflammation directly. Residual particle accumulation may have further obscured dynamic changes. Nonetheless, iron oxide imaging remains useful for therapies targeting immune cell infiltration.

### Translational potential of this study

The angiotensin II-induced AAA model in ApoE-deficient mice is well established, with 60 to 100% of animals reliably developing suprarenal aneurysms.^22^ The model mimics key pathological features of human AAA, including elastin degradation, smooth muscle cell loss, and inflammation.^22^ However, important differences exist between this model and human AAA pathophysiology.

To enhance clinical relevance, this study employed a clinical 3T MRI scanner and used molecular probes at dosages approximating those used in human applications. The elastin-specific probe’s chemical properties (structure, size, and clearance) resemble those of clinically established gadolinium-based agents, supporting the feasibility of clinical translation. Ferumoxytol, although not yet approved for routine MRI diagnostics, has been used safely in human MRI studies and is FDA-approved for treating iron deficiency anemia.^23,24^.

This molecular imaging approach offers significant clinical potential by enabling early detection of therapeutic effects before morphological changes become apparent on conventional imaging. The ability to monitor treatment response at the molecular level could facilitate personalized therapy optimization and potentially reduce the need for invasive procedures in AAA management. However, clinical validation studies will be essential to establish the safety and efficacy of elastin-specific molecular MRI in human subjects.

### Study limitations

The exclusive use of male mice necessitated by the low incidence of AAA formation in females under angiotensin II infusion (approximately 20%), limits the generalizability of our findings to female subjects.^25^ Furthermore, sex-specific differences in aneurysm progression may influence treatment efficacy and should be addressed in future studies. In addition, the dosing regimen used in this murine model represents an experimental approach that cannot be directly translated to clinically relevant human dosing without further pharmacokinetic and dose-translation studies. To minimize animal usage in this exploratory study, the saline-treated control group received alternating subcutaneous (s.c.) and intraperitoneal (i.p.) injections. As we did not expect significant differences between these two administration routes in a sham treatment, this approach was chosen to generate a versatile control group potentially applicable to future studies involving subcutaneous treatment administration. Furthermore, the suprarenal localization of AAAs in this murine model does not align with the typical infrarenal location observed in humans.^22,26^.

The regulatory status of the molecular probes represents another limitation. While ferumoxytol has been employed in clinical MRI studies, it lacks approval for routine diagnostic use. Similarly, the elastin-specific probe requires extensive safety and efficacy testing before clinical application.

Finally, the short follow-up period limits the statistical power and long-term assessment of treatment effects. A confirmatory study with a larger cohort and extended timepoints will be essential to validate these exploratory findings and establish the reproducibility of the molecular imaging approach. In addition, a study design including weekly end points would provide a more detailed characterization of disease progression and treatment response over time, while enabling closer correlation between longitudinal in vivo imaging findings and corresponding ex vivo analyses. Future confirmatory studies with larger cohorts should aim to capture the full spectrum of treatment responses, including partial and non-responders, to better reflect clinical heterogeneity.

## Conclusion

This study demonstrates that elastin-specific molecular imaging enables sensitive, non-invasive monitoring of therapeutic effects of cyclodextrin in an AAA model. In contrast, iron oxide particles showed limited utility for monitoring cyclodextrin therapy effects, due to the lack of observable changes in the inflammatory response. These findings underscore the critical importance of matching molecular imaging probes to specific therapeutic mechanisms and support the continued development of tailored imaging strategies for individualized monitoring of AAA therapies. Our results highlight the potential of integrating molecular biology with clinical imaging to advance precision medicine in vascular disease management.

## Methods

### Ethical statement

All animal procedures and care were conducted in compliance with the Directive 2010/63/EU of the European Parliament and of the Council of 22 September 2010 on the protection of animals used for scientific purposes and the local Guidelines and Provisions for Implementation of the Animal Welfare Act. The study protocols received approval from the competent authority of the Berlin State Office for Health and Social Affairs, LAGeSo Berlin (G0017/22). Additionally, this study was performed in compliance with the ARRIVE guidelines (Animal Research: Reporting of In Vivo Experiments).

### Animal experiments

Male apolipoprotein-E knockout (ApoE^−/−^) (B6.129P2-ApoE^tm1Unc^/J) mice at the age of 8–12 weeks were used for this study (25–31 g at the time of intervention). Mice were sourced from the Research Institute of Experimental Medicine at Charité Berlin from the same strain but at different times/generations.

**Fig. 5:**
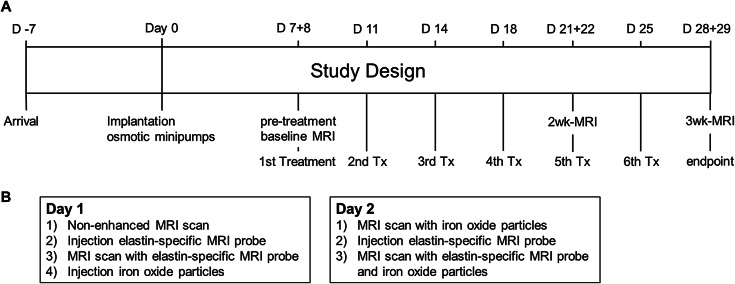
Study design for molecular MRI monitoring of cyclodextrin therapy in a murine abdominal aortic aneurysm model. **A**, Mice received angiotensin-II via osmotic minipumps to induce AAAs and were assigned to either cyclodextrin or saline treatment starting on day 7, after confirmation of aneurysm formation. Treatment (Tx) was administered twice weekly. MRI scans were performed at three timepoints: day 7/8 (pre-treatment baseline-MRI), day 21/22 (2wk-MRI), and day 28/29 (3wk-MRI), day 29 marking the study endpoint. **B**, Each MRI timepoint included two consecutive scan days with different probe combinations, as shown in the schematic.

To induce aneurysms an osmotic minipump (ALZET^®^ Model 2004, DURECT Corporation, Cupertino, CA, USA) was implanted subcutaneously in the dorsal neck region (*n* = 41). The osmotic minipumps continuously delivered angiotensin II at a rate of 1,000 ng/kg/min for 28 days.^22^ MR imaging was performed longitudinally at 1 (pre-treatment baseline-MRI), 3 (2wk-MRI) and 4 (3wk-MRI) weeks after aneurysm induction (Fig. 5A). One week after AAA induction, during the initial MRI (pre-treatment baseline MRI) animals with an AAA were identified. An AAA was defined as an increase of the aortic diameter of 50%, as observed on MRI. Animals without an AAA were excluded (*n* = 5) and 4 animals did not reach the pre-treatment baseline-MRI timepoint (*n* = 4). Following randomization, treatment was then initiated with either 2-hydroxypropyl-β-cyclodextrin (*n* = 16 cyclodextrin group) or saline (*n* = 16, saline group). At each time point, mice underwent MR imaging prior to and after the administration of the elastin-specific probe and iron oxide particles. After 4 weeks, the mice were euthanized, and the aortas were excised. Twelve animals did not reach the study endpoint, resulting in *n* = 10 animals per group. According to the defined inclusion and exclusion criteria (see Supplemental Material), only animals with a therapeutic effect were included in the cyclodextrin group, resulting in a group size of *n* = 8 (cyclodextrin group) and *n* = 10 (saline group). As the primary aim of this study was to evaluate the suitability of molecular probes for monitoring therapy-induced changes, we aimed to reproduce a therapeutic response and assess its detectability by molecular imaging. In this initial exploratory study, different stages of therapeutic response were not systematically investigated. For further information see Supplemental Material the inclusion and exclusion criteria.

### Anesthesia

Animals were anesthetized during the implantation of the osmotic minipumps and MRI procedures. Anesthesia was achieved by intraperitoneal injection of medetomidine (500 µg/kg body weight), fentanyl (50 µg/kg body weight), and midazolam (5 mg/kg body weight). A single administration was sufficient for the duration of the procedures, and no additional doses were required. To prevent corneal drying, the animals received dexpanthenol eye ointment and were placed on a heat pad during anesthesia to maintain body temperature. Anesthesia was fully antagonized by a single intraperitoneal administration of atipamezole (750 µg/kg body weight), naloxone (1200 µg/kg body weight), and flumazenil (500 µg/kg body weight). Following anesthesia thermal support was provided to the animals using an infrared light lamp to assist with temperature regulation during the recovery.

### Implantation of osmotic minipump

To implant the osmotic minipump, the dorsal neck region was shaved and disinfected. A small transverse skin incision, approximately 1 centimeter in length, was made and the minipump was implanted subcutaneously. The incision was closed using 3 to 4 sutures (USP 6/0 Vicryl™, Ethicon^®^, Norderstedt, Germany). During osmotic minipump implantation, animals received carprofen (5 mg/kg body weight, administered subcutaneously) to provide analgesia until they began consuming metamizole-infused drinking water post-procedure. Post-operative analgesia was administered via drinking water infused with metamizole (1.25 mg metamizole/ml drinking water), for the following three days.

### Molecular probes

#### Gadolinium-based elastin-specific contrast agent

To assess the molecular composition of the aortic wall, two different molecular probes were used. To visualize the quantity of elastic fibers, a gadolinium-based elastin-specific MRI probe (Lantheus Medical Imaging, North Billerica, MA, USA) was used. An estimated clinical dose of 0.2 mmol/kg (dissolved in saline) was administered in this study.

#### Iron oxide–based macrophage-specific probe

To visualize macrophage infiltration ferumoxytol (Feraheme^®^, AMAG Pharmaceuticals, Waltham, MA, USA) was used.^27^ A clinical dose of 4 mg Fe/kg body weight was administered.

#### MRIs

For MR imaging, a 3.0 Tesla MR scanner (MAGNETOM Lumina, Siemens, Erlangen, Germany) and a 4-channel receive coil array for mouse body applications (Mouse Heart Array, P-H04LE-030, Version 1, Rapid Biomedical, Rimpar, Germany) were used. MRI investigation consisted of two MRI sessions on two consecutive days. On day one, the animals were scanned prior to (non-enhanced MRI) and after intravenous (i.v.) administration of the elastin-specific contrast agent (elastin-specific contrast agent-enhanced scan) (Fig. 5B). Subsequently, iron oxide particles were administered i.v. (4 mg Fe/kg body weight). On day two, approximately 24 h following iron oxide particle application, another MRI session was performed prior to (iron oxide-enhanced scan) and following administration of the elastin specific molecular probe (dual iron oxide+elastin-enhanced scan).

#### Treatments

Initially, *n* = 16 animals received a therapeutic treatment with cyclodextrin (cyclodextrin group). These mice received intraperitoneal (i.p.) injections of cyclodextrin at a dose of 2 g/kg body weight twice weekly, starting after the first MRI session (week 1) and continuing until euthanasia (week 4). The dosing regimen was based on previously published work by Lu et al. (2020), who demonstrated therapeutic efficacy of cyclodextrin in a murine abdominal aortic aneurysm model using this schedule.^14^ Cyclodextrin powder (H107-5G, (2-hydroxypropyl)-β-cyclodextrin, Sigma-Aldrich, St. Louis, Missouri, USA) was freshly dissolved in 0.15 ml saline prior to each administration. As a control for the therapeutic effects of the cyclodextrin treatment, *n* = 16 mice received 0.15 ml saline twice weekly via alternating subcutaneous (s.c.) and i.p. injections (saline group). An alternating administration route was used to reduce the number of animals required for this type of study. As there is no expected difference in the effect on aneurysm progression between subcutaneous and intraperitoneal administration of saline, we did not anticipate any influence of the difference in administration route on the saline group. For this reason, this group could also be used as a control group for treatments administered subcutaneously in the future. This was suggested to be applicable in an exploratory context.

#### Euthanasia and sample collection

Euthanasia was performed via cervical dislocation under deep anaesthesia (as described above), followed by exsanguination. The aorta was perfused with saline before excision of the suprarenal aorta including the right and left renal arteries. The sample was immediately imbedded in OCT compound (Tissue-Tek^®^ O.C.T. Compound, Sakura Finetek Europe (SFE), Alphen aan den Rijn, Netherlands) and frozen at -20 °C to prevent thawing during future preparations.

#### MRI data analysis

The MRI data were analyzed to assess the aneurysm morphology, the contrast-to-noise ratio increase (CNR increase), and the relative signal reduction (RR). MR images were evaluated using Horos™ (Version 4.0.1, Horos Project, Geneva, Switzerland). Aneurysm morphology was evaluated by measuring the diameter, area, and circumference in the section displaying the largest expansion of the aorta. The maximum aortic diameter was recorded as a readily accessible parameter. However, due to the presence of thrombus and the often irregular, non-circular morphology of aneurysmal aortas, diameter alone was considered insufficient to accurately reflect aneurysm size. Therefore, quantitative analysis was primarily based on cross-sectional area measurements. Aortic area and circumference were determined by manually delineating a polygonal region of interest (ROI) along the vessel boundary. The maximum diameter was additionally defined as the longest linear distance across the vessel within the same cross-section. Three-dimensional (3D) reconstructions were generated for illustrative purposes only and were not used for quantitative analysis. The models were created using Horos™ based on MRI datasets. For this purpose, the vessel, including lumen and thrombus, was manually delineated as a region of interest (ROI) across consecutive image slices. These segmentations were subsequently combined to generate a three-dimensional representation of the aneurysm morphology. For the CNR (Eq. 1) and RR (Eq. 3) calculations the region of interest (ROI) was defined as the entire vessel wall including possible thrombus but excluding the lumen. Blood signal intensity was measured using a ROI defined in the lumen of the vessel. Image noise was quantified using a ROI in the background anterior to the animal. The measurement was done in three cross-sections: the one showing the largest aneurysmal expansion, one section preceding it, and one following it. In order to ensure the same localization on different MRI days, the kidneys and renal arteries were used as a fiducial marker. In T1-weighted sequences, the elastin-specific probe was evaluated using the CNR increase.^12^ Because of a wide span of CNR values prior to the elastin-specific probe application (range from 5.58 to 18.1), the relative CNR increase was used to evaluate the effect of this molecular probe in the individuals. The relative CNR increase was defined as the factorial increase of the CNR observed before and after the administration of the elastin-specific contrast agent (Eq. 2). It is expressed as a factor representing the proportional change of the CNR due to the application of the elastin-specific probe.

1 $$CNR=\frac{\text{combined vessel wall and aneurysmal signal intensity} - \text{blood signal intensity}}{\text{standard deviation of the image noise}}$$

2 $$\text{CNR increase}=\frac{\text{CNR post elastin-specific probe}}{\text{CNR prior elastin-specific probe}}$$

In T2*-weighted sequences the influence of iron oxide particles was measured using the relative reduction of the MR signal (Eq. 3).^28^

3 $$RR=\frac{\left(\text{signal intensity pre iron oxide particles}-\text{signal intensity post iron oxide particles}\right)}{\text{signal intensity pre iron oxide particles}}$$

#### Histological staining

For histological analyses, one representative section per animal was analyzed. 10 μm sections were used for Miller´s Elastica van Gieson stain (EvG), Perls Prussian blue Stain and immunofluorescence. The stained samples were captured and examined using a digital microscope (Keyence BZX-800 Viewer and Analyzer, Osaka, Japan). To evaluate the vessel morphology a computer-assisted image analysis was used (ImageJ software, Version 1.54, National Institute of Health, Bethesda, MD, USA). Images were calibrated by setting a spatial scale based on known pixel-to-micrometer conversion factors prior to morphological measurements. In addition to morphological measurements the proportion of elastic fibers and iron content within the aortic wall was measured using a digital image analysis (Keyence BZX-800 Analyzer, Version 1.1.1.8, Osaka, Japan). The content of a target was measured as a percentage of the stained area.

#### Immunofluorescence staining

For immunofluorescence staining, the sections were prepared and fixated as described in the supplemental material. Immunofluorescence staining was performed to evaluate the expression of CD68 (Rat anti-mouse CD68 antibody, clone FA-11, MCA1957, Bio-Rad, Hercules, CA, USA), TFEB (Rabbit anti-mouse TFEB antibody, 13372-1-AP, Proteintech, Rosemont, IL, USA) and transgelin (Rabbit anti-mouse transgelin/SM22 antibody, 10493-1-AP, Proteintech, Rosemont, IL, USA). CD68 was used to visualize macrophages in the aortic wall, TFEB to evaluate the effect of cyclodextrin as a TFEB activator, while transgelin served as a marker for smooth muscle cells in the aortic wall. To photograph and analyze the stained samples, a digital microscope with its associated software was used (Keyence BZX-800 Viewer and Analyzer, Osaka, Japan). The target’s content was measured as a percentage of the stained area.

#### Western blot

For Western blot analyses, lysates (cyclodextrin group: *n* = 5, saline group: *n* = 10) were prepared as described in the supplementary. In the cyclodextrin group, only 5 of the originally collected 8 samples could be used, as insufficient tissue remained from 3 samples due to their small size for reliable analysis. Western blot analyses were conducted on a Jess Automated Western Blot System (Bio-Techne, Minneapolis, MN, USA). Target proteins analyzed included Mac2 (Rabbit anti-Mouse Mac2 antibody, Dilution 1:10, 14979-1-AP, Proteintech, Rosemont, IL, USA) and elastin (Rabbit anti-Mouse Elastin antibody, Dilution 1:20, bs-1756R, Bioss, Woburn, MA, USA). Cartridge preparation followed the manufacturer´s protocol, and analysis was performed using the Compass for Simple Western software (Version 6.3.0, Bio-Techne, Minneapolis, MN, USA). Protein expression levels were quantified (corrected area after protein normalization) and compared between treatment groups.

#### Laser ablation–inductively coupled plasma–mass spectrometry (LA-ICP-MS)

10 μm cryosections from the samples of *n* = 3 per group were used for LA-ICP-MS. For the analysis of Gd and Fe accumulation of the sections the imageBIO266 laser ablation system (Elemental Scientific Lasers, Bozeman, MT, USA) was used.

### Statistical analysis

The statistical analysis was conducted in an exploratory framework appropriate for a pilot investigation. The analysis was performed using IBM SPSS Statistics for Windows (Version 30.0.0.0, IBM Corp., Armonk, NY, USA). Continuous variables are reported as means ± standard deviations. Differences between experimental groups and timepoints are described using conventional inferential statistics and corresponding effect sizes. Two-tailed p-values are interpreted in an exploratory context and are not intended to support confirmatory conclusions. Statistical significance was defined as *p* < 0.05 and is interpreted descriptively.

In addition to formal normality testing, the primary outcome variable is expected to be approximately normally distributed in the underlying population, based on its physiological nature and previous reports in comparable experimental models. Accordingly, and in the absence of extreme outliers, parametric methods were considered appropriate for the primary analyses. Intra-group differences over time were assessed using paired t-tests, and inter-group comparisons were performed using unpaired t-tests. Effect sizes were estimated using Cohen’s d (d), and relationships between variables were assessed with Pearson’s correlation coefficient (r). All parametric results are reported in the format p = X.XXX, d = X.XX and Pearson’s correlation coefficient (r) was reported as r = X.XX, p = X.XX. Correlation analyses were performed using paired data from the same animals, matching in vivo measurements with corresponding ex vivo measurements on an individual basis.

To evaluate the robustness of the findings with respect to distributional assumptions, complementary non-parametric analyses were performed. These analyses yielded results that were comparable in terms of direction and interpretation to the parametric tests (details not shown).

## Supplementary Information

Below is the link to the electronic supplementary material.

Supplementary Material 1

## Data Availability

The original datasets generated during this study are available upon reasonable request.

## Abbreviations

Cyclodextrin: 2-hydroxypropyl-β-cyclodextrin
TFEB: transcription factor EB
LA-ICP-MS: laser ablation-inductively coupled plasma- mass spectrometry
ApoE^−/−^: apolipoprotein-E knockout
SPF: specific pathogen-free
IVCs: individually ventilated cages
CNR increase: contrast-to-noise ratio increase
RR: relative signal reduction
EvG: Elastica van Gieson stain
