# Supplementary material for: Molecular MRI monitoring of cyclodextrin therapy in murine abdominal aortic aneurysms
Source: Sci Rep. 2026 Jul 20;16:22666. doi: 10.1038/s41598-026-61318-8 (PMC13385629; doi:10.1038/s41598-026-61318-8)
Supplement: Supplementary file 1 — Supplementary Material 1 [file 41598_2026_61318_MOESM1_ESM.docx]

**Supplemental Material**

**Supplemental Methods**

## Randomization and Masked Evaluation

## Randomization was performed in Microsoft Excel (Microsoft, Redmond, WA, USA) using the RAND() function. Each subject was assigned an automatically generated random number between 0 and 1, and the list was subsequently sorted according to these random numbers. Participants were then allocated to the study groups in the predefined order, ensuring equal group sizes. During in vivo experiments, the operator of the studies was aware of group allocations. However, outcome assessment and data analysis were conducted in a blinded manner. Each animal and its corresponding data were assigned a unique ID to ensure that group identification remained concealed throughout the assessment and analysis of the data.

## Animal Husbandry Conditions

The animals were housed under specific pathogen-free (SPF) barrier conditions in individually ventilated cages (IVCs) with a 12-hour dark/light cycle, 55 ± 10 % humidity, and an ambient temperature of 22 ± 2 °C. Water and standard laboratory diet (complete feed for rats and mice, Cat. No. V1534-000, sniff, Soest, Germany) were provided *ad libitum*. Mice were housed in groups of up to six animals and provided with environmental enrichment items, including tubes, chew sticks, paper towels/nesting material, and shelters within their cages.

## Inclusion and exclusion criteria

Only animals with an AAA present on day 7 were included in the study. An AAA was defined as an increase of the aortic diameter of 50%, as seen on MRI. Animals without an aneurysm on day 7 were excluded. This inclusion criterion was established a priori. Only animals that completed the entire study through day 29 were considered for data analysis. In the cyclodextrin group, only those animals, where a therapeutic effect was observed, are included in the data analysis. A therapeutic effect was defined as the aneurysm not increasing by more than another 50% of the physiological diameter since the first MRI scan, resulting in a total increase of no more than 100% of the physiological diameter of the aorta. Therefore 8 out of the 10 animals of the cyclodextrin group, which underwent the 3^rd^ MRI scan are included in the data analysis. All 10 mice of the saline group that underwent the 3^rd^ MRI are included in the data analysis.

## Molecular probes

The elastin-specific probe is a low molecular-weight (856 g/mol) compound^1^. *Ex vivo* analysis revealed a longitudinal relaxivity of 8.65 ± 0.42 mM^−1^ s^−1^ at 3 T for the agent bound to murine aortas^1^.

Ferumoxytol exhibits an extended blood-pool phase, characterized by a plasma half-life of 14 to 21 hours in humans, followed by delayed intracellular uptake^2^. MR imaging for macrophage visualization was performed 24 hours after injection to allow cellular uptake

## MRI procedure and sequences

During MR imaging the animals were under general anesthesia, which was antagonized after the MRI session. Animals were placed in a prone position on the MRI patient table. For the intravenous application of the molecular probes a venous port, using a small diameter tube with an attached 30 G cannula, was placed in the tail vein. The elastin-specific probe (0,2 mmol/kg body weight) and ferumoxytol (4 mg Fe/kg body weight) was each diluted in 0,1 ml saline prior to injection.

To obtain an overview of the recording field, two localizer sequences were initially employed. A three-dimensional (3D) time-of-flight angiography was conducted in transverse orientation to visualize the abdominal aorta, applying the parameters listed below: field of view (FOV) of 200 × 200 mm, a matrix of 752 × 752, resolution of 0.3 × 0.3 × 0.5 mm³, 40 slices, slice thickness of 0,5 mm, repetition time (TR) of 41.6 ms, echo time (TE) of 3.84 ms, flip angle of 90°, and a bandwidth of 266 Hz/Px (morphological measurements). After that a high-resolution 3D IR FLASH sequence was used to visualize the signal of the aortic wall in a T1-weighted MRI with following parameters: FOV of 57 × 57 mm, matrix of 416, resolution of 0.1 × 0.1 × 0.4 mm³, 56 slices, slice thickness of 0.4 mm, TR of 1061.0 ms, TE of 7.38 ms, TR between subsequent inversion recovery (IR) pulses 1000 ms, inversion time (TI) of 490 ms, flip angle of 30°, and a bandwidth of 125 Hz/Px (CNR prior elastin-specific probe, equation 2). To investigate the effect of ferumoxytol on the signal of the aortic wall a T2*-weighted MRI sequence was used with the following parameters: FOV of 200 × 200 mm, matrix of 832 × 832, resolution of 0.2×0.2×0.4 mm³, 40 slices, slice thickness of 0.4 mm, TR of 17.0 ms, TE of 7.38 ms, flip angle of 20°, and a bandwidth of 180 Hz/Px (RR measurements on day 1 and day 2 of the consecutive imaging days, equation 3). After the administration of the ESMA another T1-weighted sequence was used with those parameters: FOV of 57 × 57 mm, matrix of 384 × 384, resolution of 0.1 × 0.1 × 0.4 mm³, 56 slices, slice thickness of 0.4 mm, TR of 1019.7 ms, TE of 7.16 ms, TR between subsequent IR pulses 1000 ms, TI of 230 ms, flip angle of 30°, and a bandwidth of 130 Hz/Px (CNR post elastin-specific probe, equation 2).

## Histological staining

From the OCT-embedded samples, 10 µm cryosections were cut at -20 °C using a Leica cryostat (Leica CM3050s Cryostat, Leica Biosystems, Nussloch, Germany) and mounted onto SuperFrost Plus adhesion slides (Epredia™ SuperFrost Plus™ Slides, Cat. No. J1810AMNZ, Epredia, Portsmouth, NH, USA). Prior to staining, the frozen sections were prepared according to this fixation protocol. The sections were first allowed to thaw at room temperature for 30 minutes, then fixed in ice-cold acetone for 5 minutes. Then the sections were air-dried at room temperature for 30 minutes. Subsequently, the slides were washed three times in phosphate-buffered saline (PBS) (ROTI^®^Fair PBS 7.4, Cat. No. 111.2, Carl Roth, Karlsruhe, Germany) for 3 minutes each.

For the Elastica van Gieson stain, slides were incubated with potassium permanganate (Morphisto, Offenbach, Germany) for 5 minutes and rinsed in tap water for 30 seconds. Next the sections were incubated in oxalic acid for 1–2 minutes, until decolorization, and washed in distilled water for 30 seconds followed by ethanol for 30 seconds. Then the sections were incubated in Miller's elastin staining solution (VWR, Radnor, PA, USA) for 45 minutes and rinsed with ethanol until staining residues were removed. Following this, the slides were incubated in Van Gieson Pichrofuchsin (Morphisto, Offenbach, Germany) staining solution for 5 minutes and washed twice in distilled water for 2 minutes each. The sections were then dehydrated using an ascending alcohol series, mounted with ROTI^®^Mount mounting medium (ROTI^®^Mount, Carl Roth, Karlsruhe, Germany), and covered with a coverslip.

For iron staining, Perls Prussian blue stain was applied. The sections were incubated in a 1:1 mixture of potassium ferricyanide (2%) and hydrochloric acid (2%) for 35 minutes. Following incubation, the slides were washed three times with distilled water for 2 minutes each. Then the samples were incubated in nuclear red (Morphisto, Offenbach, Germany) for 5 minutes and washed three times with distilled water for 2 minutes each. After dehydration through an ascending series of alcohols, the slides were mounted with ROTI^®^Mount mounting medium (ROTI^®^Mount, Carl Roth, Karlsruhe, Germany), and covered with a cover slip.

To measure the proportion of elastic fibers or iron, the area of the target was measured against the total area of the sample using the Hybrid Cell Count analysis application.

## Immunofluorescence

Prior to dilution, the primary antibodies were centrifuged and vortexed. All antibodies were diluted 1:100 in Dako REAL Antibody Diluent (Dako, Agilent, Carpinteria, CA, USA). On each sample one primary antibody was applied. The slides were incubated overnight in a humid chamber with deionized water at 4°C. After incubation, the slides were washed twice with PBS-TWEEN (PBS containing 0.05% TWEEN^®^ 20 (Cat. No. P1379, Sigma-Aldrich^®^, St. Louis, MO, USA)) for 2 minutes each. The secondary antibodies were centrifuged and vortexed before dilution in Dako REAL Antibody Diluent. For CD68 staining the Rabbit anti-Rat - Alexa Fluor 594 secondary antibody (Rabbit anti-Rat IgG (H+L) Cross-Adsorbed Secondary Antibody, Alexa Fluor 594, A-21211, Invitrogen, Carlsbad, CA, USA) was used at a 1:200 dilution. For TFEB and transgelin, the Chicken anti-Rabbit - Alexa Fluor 594 secondary antibody (Chicken anti-Rabbit IgG (H+L) Cross-Adsorbed Secondary Antibody, Alexa Fluor 594, A-21442, Invitrogen, Carlsbad, CA, USA) was used at a 1:500 dilution. The secondary antibodies were incubated for 1 hour at room temperature darkened in a humid chamber. The slides were washed two times with PBS-TWEEN for 2 minutes each. For mounting and counterstaining, 1–2 drops of Fluoromount with DAPI (Roti®-Mount Fluor Care, Carl Roth, Karlsruhe, Germany) were applied to each slide. The slides were left in a darkened humid chamber overnight to dry the mounting solution before analyzing them.

To evaluate the proportion of each antigen within the aortic wall, the area of the secondary antibody signal was quantified relative to the area of DAPI-stained background using the Hybrid Cell Count module.

## Western Blot

The cooled samples (cyclodextrin group: n=5, saline group: n=10) were homogenized using a homogenizer in a solution containing lysis buffer (per 1L: 10 g SDS, 1.576 g Tris-HCl, 420 g urea, 100 ml glycerol, and H2O) and Halt™ Protease and Phosphatase Inhibitor Cocktail (Cat. No. 78440, Thermo Scientific™, Waltham, MA, USA). The homogenized samples were vortexed and then placed on a shaker at 100 rpm for 2 hours at 4°C. Samples were centrifuged at 12,000 rpm for 20 minutes at 4°C. Protein concentration was determined using the Pierce™ BCA Protein Assay Kit (Cat. No. 23225, Thermo Fisher Scientific, Waltham, MA, USA) via microplate procedure. The target concentration was 1.25 mg/mL. When necessary, protein concentrations in the solutions were increased using a Pierce™ Protein Concentrator PES, 3K MWCO (Cat. No. 88515, Thermo Fisher Scientific, Waltham, MA, USA).

Following kits were used: EZ Standard Pack for 12–230 kDa, 25-Capillary Cartridge (12–230 kDa) (Cat. No. PS-ST01EZ-8, Bio-Techne, Minneapolis, MN, USA), Protein Normalization Module (Cat.No. DM-PN02, Bio-Techne, Minneapolis, MN, USA), and Anti-Rabbit Detection Module (Cat. No. DM-001, Bio-Techne, Minneapolis, MN, USA). Protein separation and immunodetection were performed using the automated Simple Western system. Protein samples were prepared at a target concentration of 1.25 mg/mL. Equal amounts of protein were loaded into each capillary according to the manufacturer’s instructions. The separation time was 25 min at a separation voltage of 375 V. Total protein normalization was applied using the Protein Normalization Module of the manufacturer. The system automatically calculates normalized signal intensities based on the total protein content within each capillary. Raw peak area values were converted into normalized values using Compass for Simple Western software (ProteinSimple), without manual intervention. Protein normalization was carried out for 25 min, followed by antibody incubation steps including 5 min antibody diluent time, 30 min primary antibody incubation, and 30 min secondary antibody incubation.

## Laser Ablation–Inductively Coupled Plasma–Mass Spectrometry

The laser ablation system was coupled to an iCAP TQ ICP-MS (Thermo Fisher Scientific, Bremen, Germany) using a dual concentric injector (DCI, Elemental Scientific Lasers, Bozeman, MT, USA). A line-by-line scan was performed with a spot size of 5 μm and a scan speed of 50 μm/s. The laser energy was set to 3 J/cm2 with a shot frequency of 100 Hz. To transport the ablated sample into the ICP-MS system, a helium gas flow with a flow rate of 1400 ml/min was applied.

The interface of the ICP-MS system includes a sampler and a skimmer each made of nickel. The plasma power was set to 1550 W with an auxiliary gas flow of 0.80 L/min and a cool gas flow of 14 L/min. The nebulizer gas flow as well as the detector settings were tuned daily.

For the detection of the elements of interest, oxygen was used as a reaction gas. The elements were recorded as the following ions: ^158^Gd^16^O+, ^56^Fe^16^O+, ^66^Zn+, ^65^Cu+ and ^31^P^16^O+. The dwell time was set to 20 ms for each ion. Processing of the images for the localization and quantification of the elements in the section was carried out with the inhouse-developed software Imajar 3.64 (written by Robin Schmid).

For the quantification of iron and gadolinium, an external calibration with matrix-matched standards made of gelatin was utilized. To spike the gelatin with different analyte concentrations, iron sulfate and gadolinium chloride were used. Eleven gelatin standards (10% w/w) were prepared. The spiked gelatin standards ranged from 0.1 µg/g to 1000 µg/g for iron and 0.01 µg/g to 500 µg/g for gadolinium. The standards were cut into 10 μm thin sections using a Cryostar NX70 cryostat (Thermo Fisher Scientific, Waltham, MA, USA). The limit of detection (LOD) was 33 µg/g and the limit of quantification (LOQ) was 100 µg/g for iron and a LOD of 0.4 µg/g and a LOQ of 1.1 µg/g were calculated for gadolinium. The LODs and LOQs were calculated with the 3σ- and 10σ-criteria^3^.

## Statistical Analysis

Initially, the data were examined separately by group to explore distributional patterns and identify potential outliers. Normality of continuous variables was assessed using the Shapiro-Wilk test and visual inspection. Only mild outliers were detected, and these were retained in the analysis. To analyze intra-group effects over time, paired t-tests were used to compare measurements from individual animals at different time points. For inter-group comparisons at each time point, the assumption of homogeneity of variances was tested first. If variances were equal, an independent samples t-test was performed. In cases of unequal variances, Welch’s t-test was applied. The effect size was calculated using Cohen’s d (d).

Additionally, corresponding non-parametric tests (Wilcoxon signed-rank tests for paired comparisons, Mann–Whitney-U-tests for independent group comparisons) were performed. These analyses produced results consistent with the parametric analyses with respect to effect direction and overall interpretation; therefore, only the parametric results are reported in detail in the main text.

**Supplemental Results**

## Overview of the Study Course

41 animals underwent the AAA induction procedure (n=41). Four animals died or were euthanized between implantation of the osmotic minipump and the baseline MRI scan: three due to aneurysm rupture and one due to poor general condition. One week after AAA induction, MR imaging confirmed an aortic dilatation of >50% in n=32 animals. N=5 animals did not develop an AAA and were excluded from the study*.*

Following confirmation of aneurysm formation, animals were randomly assigned to two treatment groups: cyclodextrin (n=16) and saline (n=16). Out of these 32 animals, n=3 animals died due to aortic rupture between day 7 and day 29 after AAA induction (cyclodextrin group=2, saline group=1). N=9 animals did not reach the study endpoint: n=3 due to murine urologic syndrome, n=1 due to exclusion because of a large aneurysm with high rupture risk, n=1 due to poor general condition, and n=4 due to unknown causes. Only animals that completed the full study period up to day 29 were included in the initial analysis, resulting in a reduced group size of n=10 per group.

Two animals (n=2) in the cyclodextrin group displayed aneurysm progression exceeding 100% of the physiological diameter of the aorta and were therefore classified as non-responders, and excluded from subsequent analyses (see Supplemental Material for inclusion and exclusion criteria). Thus, the final sample size for efficacy analyses included n=8 in the cyclodextrin group and n=10 in the saline group.

## Overview of reported data

In addition to Table 1, all reported data are presented collectively in one table (Table S1).

**Supplementary Figures**

Supplementary Figure1


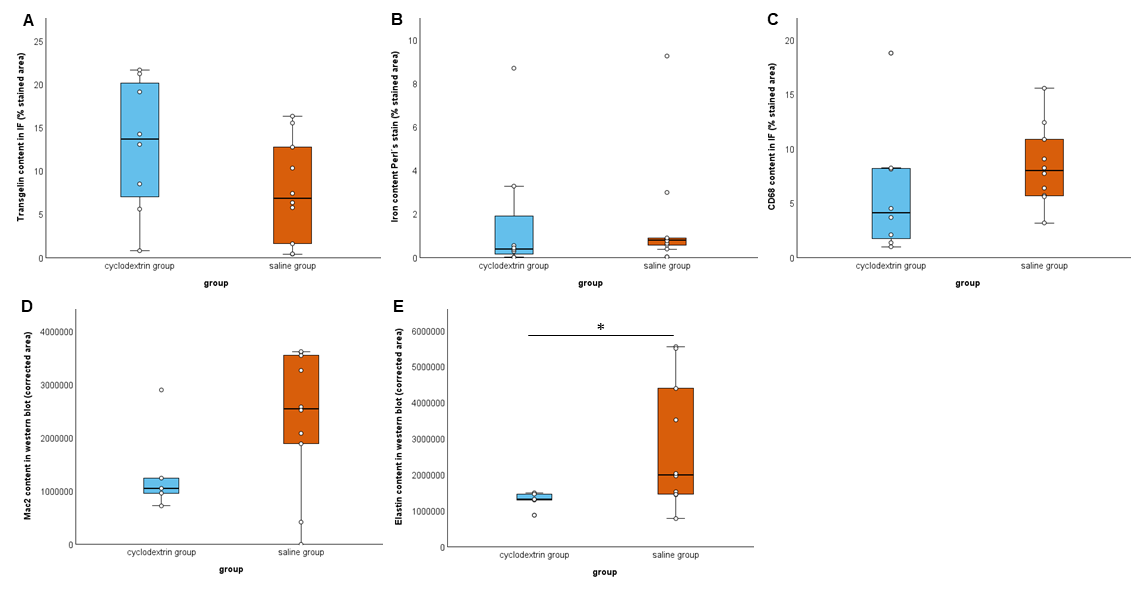


Supplementary Figure 1. Ex vivo measurements

**A**, Transgelin expression assessed by immunofluorescence staining, quantified as the percentage of stained area. **B,** Iron deposition evaluated by Perls’ staining, quantified as the percentage of stained area. **C**, CD68-positive area assessed by immunofluorescence staining, expressed as the percentage of stained area. Panels A to D consist each of n = 8 biological replicates in the cyclodextrin group and n = 10 biological replicates in the saline group. **D**, Mac2 protein expression analyzed by Western blot, quantified as corrected area. **E**, Elastin protein expression analyzed by Western blot, presented as corrected band area, shows significant differences (cyclodextrin group: 1.29 × 10⁶ ± 0.25 × 10⁶ compared to saline: 2.83 × 10⁶ ± 1.78 × 10⁶, Welch’s t-test: p = 0.025). Western Blot measurements consist of n = 5 biological replicates in the cyclodextrin group and n = 10 biological replicates in the saline group.

Statistical significance: *ns = not significant (p ≥ .05), * p < .05, ** p < .01, *** p < .001.*

Supplementary Figure 2


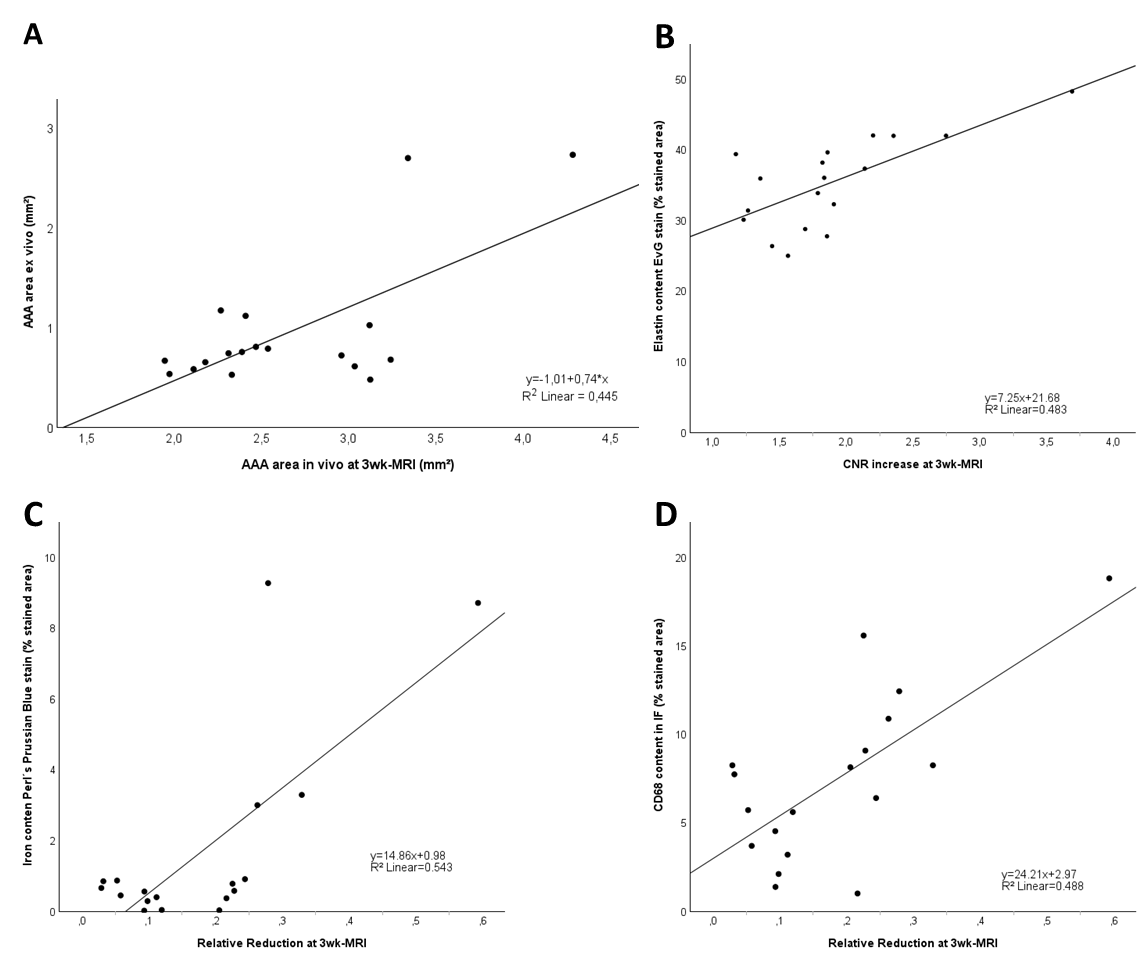


Supplementary Figure 2. Correlation of *in vivo* MRI measurements with *ex vivo* histological and morphological data.

**A**, Strong correlation between *in vivo* cross-sectional AAA area from MRI and ex vivo area measurements (r = 0.667, p = 0.003) (n=20 biological replicates). Statistical analysis was performed using Pearson’s correlation coefficient (r). **B**, *In vivo* contrast-to-noise ratio (CNR) increase after elastin-specific probe application correlates significantly with ex vivo elastin content quantified from Elastica van Gieson staining (r = 0.695, p = 0.001) (n=18 biological replicates). **C,** *In vivo* T2* signal reduction correlated with iron content detected via Perls’ Prussian Blue staing (r = 0.737, p < 0.001) (n=18 biological replicates). **D**, T2* signal reduction also significantly correlated with the number of CD68-positive macrophages identified by immunofluorescence (r = 0.699, p = 0.001) (n=18 biological replicates). These correlations support the validity of dual molecular MRI for assessing extracellular matrix integrity and inflammation in AAA.

Table S1. Overview of reported data

| parameter | cyclodextrin group | saline group |
| --- | --- | --- |
| TFEB stained area (IF) (% stained area) | 11.28±8.28 | 4.09±3.89 |
| transgelin stained area (IF) (% stained area) | 13.05 ± 7.61 | 7.72 ± 5.94 |
| elastin stained area (EvG) (% stained area) | 31.27 ± 3.68 | 38.59 ± 6.29 |
| iron stained area (Perls stain) (% stained area) | 1.72 ± 3.02 | 1.74 ± 2.76 |
| CD68 stained area (IF) (% stained area) | 5.99 ± 5.88 | 8.49 ± 3.66 |
| Mac2 content (WB) (corrected area) | 1.38 × 10⁶ ± 0.87 × 10⁶ | 2.35 × 10⁶ ± 1.29 × 10⁶ |
| elastin content (WB) (corrected area) | 1.29 × 10⁶ ± 0.25 × 10⁶ | 2.83 × 10⁶ ± 1.78 × 10⁶ |

**References**

1. Makowski MR, Wiethoff AJ, Blume U, Cuello F, Warley A, Jansen CH, Nagel E, Razavi R, Onthank DC, Cesati RR, et al. Assessment of atherosclerotic plaque burden with an elastin-specific magnetic resonance contrast agent. *Nat Med*. 2011;17:383–388. doi: 10.1038/nm.2310

2. Toth GB, Varallyay CG, Horvath A, Bashir MR, Choyke PL, Daldrup-Link HE, Dosa E, Finn JP, Gahramanov S, Harisinghani M, et al. Current and potential imaging applications of ferumoxytol for magnetic resonance imaging. *Kidney Int*. 2017;92:47–66. doi: 10.1016/j.kint.2016.12.037

3. Rodriguez LC, Campana AMG, Linares CJ, Ceba MR. Estimation of Performance-Characteristics of an Analytical Method Using the Data Set of the Calibration Experiment. *Anal Lett*. 1993;26:1243–1258. doi: Doi 10.1080/00032719308019900
